# Editorial: Amino acids in intestinal growth and health

**DOI:** 10.3389/fnut.2023.1172548

**Published:** 2023-03-22

**Authors:** Yun Ji, Yongqing Hou, François Blachier, Zhenlong Wu

**Affiliations:** ^1^State Key Laboratory of Animal Nutrition, College of Animal Science and Technology, China Agricultural University, Beijing, China; ^2^Hubei Key Laboratory of Animal Nutrition and Feed Science, Wuhan Polytechnic University, Wuhan, China; ^3^The National Institute for Agriculture, Alimentation and Environment, Nutrition Physiology and Ingestive Behavior Unit, Université Paris-Saclay/AgroParistech/INRAE, Paris, France

**Keywords:** amino acids, gut health, immunity, intestine, gut microbiota

The intestinal epithelium serves not only as a selective barrier with an ability to protect host against pathogens invasion and act as a major site for nutrient absorption and metabolism, but also participates to the immune system in animals and humans. In addition to the structural and functional homeostasis and microbial balance of the gut, the central determinant for intestinal health is associated with interactions between nutrients, trillions of intestinal microbes, and the intestinal epithelium (1). Disruption of gut homeostasis and breakdown of the barrier will eventually lead to multiple gastrointestinal diseases.

Besides serving as substrates for protein synthesis, amino acids, and various derived metabolites play a vital role in the regulation of intestinal growth and development, immunity, maintenance of redox homeostasis, and the gut microbiota (2). Functional amino acids, including glutamine, arginine, glycine, glutamic acid, and tryptophan, have been reported to improve intestinal growth and ameliorate intestinal inflammatory disorders. These novel findings advance our understanding on the activities of amino acids.

We are pleased to present this Research Topic *Amino acids in intestinal growth and health*. Original and review papers in this issue focus on how dietary amino acids affect the growth, development, and immune response in mammals, notably at the intestinal level, therefore contributing to the of health of host.

Besides its primary role as a neurotransmitter, aspartate has been shown to fulfill a diverse array of other physiological functions, such as protein synthesis, hormone secretion, neurons protection, and reproductive regulation (3). It is becoming progressively apparent that macrophages play a pivotal role in preserving intestinal homeostasis and in serving as sentinels for the intestinal immune system to ward off specific infections. Aspartate is a critical precursor for the synthesis of pyrimidine and purine. Interestingly, M1 macrophages displayed changed pyrimidine metabolism, whereas M2 macrophages exhibited an alteration in aspartate metabolism (4). However, the role and mechanism of aspartate in macrophage polarization remain largely ambiguous. According to the research conducted by Wang et al., aspartate triggered metabolic reprogramming and the activation of HIF-1α (hypoxia inducible factor-1α) and NLRP3 (NOD-like receptor protein-3) inflammasome in peritoneal macrophages, hence favoring their polarization toward an M1 state. Asparagine, a derivative of aspartate, triggered cellular metabolic reprogramming and the activation of HIF-1α and inflammasome signaling, leading to an increase in interleukin-1β production from M1 macrophages. Nucleotides, however, did not exhibit this effect.

Tryptophan, phenylalanine, and tyrosine are classified as aromatic amino acids due to the presence of benzyl-based aromatic groups. Aromatic amino acids play a role in metabolic and immune processes beyond simply being the building blocks of proteins (2). Intestinal Ca^2+^-sensing receptor (CaSR), a sensor preferentially activated by aromatic amino acids, is implicated in anti-inflammatory processes in intestinal epithelium (5). However, it is uncertain whether a corresponding shift in amino acid availability occurs in response to the activation of CaSR. As demonstrated by Duanmu et al., lipopolysaccharide-induced inflammation modifies amino acid metabolism likely by changing the profiles of amino acids in serum and intestinal mucosa, and by changing the apparent ileal digestibility of amino acids in piglets. An increase in amino acid sensing and utilization was observed in piglets fed a diet supplemented with 0.16% tryptophan, 0.41% phenylalanine, and 0.22% tyrosine, which may help to meet the high demands for specific amino acids in response to an inflammatory challenge, thereby exerting anti-inflammatory benefits. The findings from this research may dictate current and future guidelines for the administration of aromatic amino acids to animals and humans suffering from inflammatory diseases in the gut.

The importance of certain functional amino acids (e.g., threonine) in aquatic species has piqued scientific interest (6). Evidence has shown that threonine promotes the development of the hepatopancreas, the proliferation and differentiation of enterocytes, and protein synthesis in fish (7, 8). Also, threonine supplementation increased whole body weight, as well as protein and lipid contents in fish. Nevertheless, the impact of threonine on immunological responses in aquatic animals remains not addressed explicitly. Study on teleost grass carp by Dong et al. examined the role of threonine on immune response. They demonstrated that threonine supplementation boosted the mRNA abundances of anti-inflammatory cytokines and suppressed NF-κB (nuclear factor kappa-B), leading to decreased pro-inflammatory cytokines. Besides, threonine was shown to regulate immunocyte biomarkers, the activities of non-specific immune active substances, and the mRNA encoding antimicrobial peptides. Furthermore, the anti-inflammatory effects and related signaling mechanisms were further validated in macrophages from head kidney of fish *in vitro*.

The specialized casein proteins of milk not only enable infants and young mammals to obtain amino acids for growing, but they also bind calcium and phosphorus for healthy bone development. The high protein and calcium content of milk comes from the presence of a variety of proteins, including A2 β-casein (9). Study from Liu et al. characterized the effect of milk with A2 β-casein type on immune, gut microbiota, and intestinal morphology using a mouse model. Their findings showed that ingestion of A2-type β-casein milk increased serum immunoglobulin E and G, improved the morphology of the small intestine, and increased abundance of *Lactobacillus*. These results indicated that supplementation of A2-type β-casein might be a potential strategy to enhance immunological function of intestine.

In conclusion, the present Research Topic presents new data regarding the relationship between amino acids and gut health. These new data ask obviously for additional studies to further reveal the underlying mechanisms that sustain these beneficial activities of amino acids under various conditions.

## Author contributions

All authors listed have made a substantial, direct, and intellectual contribution to the work and approved it for publication.
